# Methods for recording and measuring tonic GABA_A_ receptor-mediated inhibition

**DOI:** 10.3389/fncir.2013.00193

**Published:** 2013-12-05

**Authors:** Damian P. Bright, Trevor G. Smart

**Affiliations:** Department of Neuroscience, Physiology and Pharmacology, University College LondonLondon, UK

**Keywords:** tonic inhibition, GABA_A_, extrasynaptic, methods, noise

## Abstract

Tonic inhibitory conductances mediated by GABA_A_ receptors have now been identified and characterized in many different brain regions. Most experimental studies of tonic GABAergic inhibition have been carried out using acute brain slice preparations but tonic currents have been recorded under a variety of different conditions. This diversity of recording conditions is likely to impact upon many of the factors responsible for controlling tonic inhibition and can make comparison between different studies difficult. In this review, we will firstly consider how various experimental conditions, including age of animal, recording temperature and solution composition, are likely to influence tonic GABA_A_ conductances. We will then consider some technical considerations related to how the tonic conductance is measured and subsequently analyzed, including how the use of current noise may provide a complementary and reliable method for quantifying changes in tonic current.

## Introduction

Tonic inhibition is mediated by extrasynaptic GABA_A_ receptors of defined subunit composition (usually containing the α5 or δ subunits). These receptors display a high affinity for GABA that allows them to respond to the low ambient levels of GABA present in the extracellular space and generate a persistent “tonic” inhibition. Tonic conductances have been found to be important regulators of neuronal excitability *in vivo* in granule cells of the cerebellum (Chadderton et al., 2004; Duguid et al., 2012) and olfactory bulb (Labarrera et al., 2013). However, most studies of tonic inhibition have been conducted *in vitro* in acute brain slice preparations and these have allowed the identification of tonic GABA_A_ receptor-mediated conductances in all major brain areas including cortex, hippocampus, thalamus, hypothalamus and brain stem (Farrant and Nusser, 2005; Brickley and Mody, 2012). Thus, recordings of tonic inhibition have been made in many different labs under a variety of different experimental conditions, many of which are likely to impact upon the magnitude of the tonic conductance.

Furthermore, various methods are available to analyse tonic conductances. This diversity of experimental conditions and analytical methods makes comparison of tonic inhibition between studies quite difficult. In this review, we will firstly consider how different experimental conditions, including choice of experimental animal, recording temperature and solution composition, influence the tonic GABA conductance. We will then consider the available methods for measurement and analysis of the tonic conductance, including how the use of current noise may provide a complementary method for analysis of tonic inhibition.

It should be noted that inhibition is a physiological process whereby the probability of a neuron generating an action potential is reduced. Therefore, the phrase “tonic inhibition” should only be used strictly when action potentials become less likely. The action of the high-affinity extrasynaptic receptors that underlie tonic GABA_A_ conductances may of course be excitatory, depending upon both the reversal potential and the magnitude of the conductance (Farrant and Kaila, 2007; Song et al., 2011). However, here, we use the phrase “tonic inhibition” to describe the activity of extrasynaptic GABA_A_ receptors, even when the effect on action potential generation has not been quantified, since this has become widely accepted as a convention within the field.

## Experimental conditions for recording *in vitro* tonic inhibition

### Age of animal

Generally, brain slices are prepared from either mice or rats, although other animals are used in some labs. In most cases, immature animals are used since slices from younger animals seem to harbor a higher proportion of healthy cells compared to adults, and also the lack of fully developed connective tissue makes it easier to visualize and patch cells within the preparation. However, the use of juvenile animals presents other problems, since many of the systems associated with neurotransmitter release, detection and uptake, are not fully developed. Indeed, early recordings of tonic inhibition in cerebellar granule cells demonstrated a developmental increase in tonic GABA conductance over the first few postnatal weeks (Brickley et al., 1996) that mirrored the increased expression of underlying α6-containing receptors (Kaneda et al., 1995; Tia et al., 1996). Later studies investigating developmental regulation of the tonic conductances in dentate gyrus granule cells (DGGCs) and relay neurons of the ventrobasal (VB) thalamus have also shown an increasing tonic conductance over the initial postnatal period (Peden et al., 2008; Holter et al., 2010; Lee and Liou, 2013).

Interestingly, it appears that the subunit composition of the GABA_A_ receptors responsible for tonic inhibition in DGGCs may also be age-dependent, with potentially a larger α5-mediated component present in immature cells, compared with increased α4δ-GABA_A_ receptors in the adult (Glykys et al., 2008; Herd et al., 2008; Holter et al., 2010). A very recent study has examined tonic inhibition within the auditory thalamus and shown that in old rats (28–32 months), the tonic conductance displayed by neurons of the medial geniculate body (MGB) is reduced by 50% compared with young adult (3–8 months) animals (Richardson et al., 2013). Thus, the age of the experimental animal is a key factor in determining the magnitude of the tonic inhibitory conductance.

### Storage and perfusion of slices

The manner in which brain slices are stored prior to their use and perfused with solutions during recording may also be important variables in controlling tonic current amplitudes. It has been demonstrated that the tonic current recorded in hippocampal CA1 pyramidal cells is greater in slices stored in an interface chamber compared with the more commonly used submerged chamber (Glykys and Mody, 2006). It was suggested that this might be due to a decrease in GABA uptake, altered protein phosphorylation or a lower metabolic state of distress for cells stored in the interface chamber.

It is also apparent that the way in which slices are perfused with solution during recording can impact upon neuronal activity and thus may affect tonic GABA inhibition. For whole-cell recording, brain slices are usually placed on a thin glass plate and superfused with artificial cerebrospinal fluid (aCSF) i.e., the slice is submerged in recording solution. Neurons can then be visualized at high resolution using a water-immersion objective. The disadvantage of this set-up is that slices are only effectively perfused from one side and gradients for oxygen and nutrients may develop within the slice, leading to compromised neuronal activity. This can be a particular problem for experiments where high levels of neuronal activity are necessary, for instance, in the generation of network oscillations (Hajos et al., 2004; Hajos and Mody, 2009). Some improvement can be gained by using higher flow rates to improve the oxygen supply to the slice (Hajos et al., 2009).

Although not specifically addressed, perfusion rate (and hence oxygen supply) may also be important for tonic inhibition, since at higher flow rates inhibitory interneurons within the hippocampal CA3 area showed greater firing activity and CA3 pyramidal cells received a higher frequency of inhibitory synaptic input (Hajos and Mody, 2009; Hajos et al., 2009). Since tonic inhibition has been shown to be dependent upon GABA derived from synaptic release (Bright et al., 2007; Glykys and Mody, 2007b), a higher perfusion rate might be expected to be coupled to an enhanced tonic conductance. However, it should also be noted that enhanced flow rate might lead to greater wash-out of ambient transmitter within the slice so the effects of perfusion rate on tonic inhibition are not easy to predict.

### Recording temperature

Whole-cell recordings within brain slices are generally made either at room temperature (20–23°C) or at an elevated temperature, closer to physiological body temperature (37°C). However, stable recordings can be difficult to maintain at higher temperatures and therefore, intermediate temperatures are often used (30–35°C). Thus, recording temperatures can vary over a considerable range. Given the large temperature dependence of enzymatic reaction rates, it is unsurprising that changes in temperature can modulate intrinsic membrane properties and synaptic potentials in neurons (Thompson et al., 1985; Volgushev et al., 2000; Trevelyan and Jack, 2002; Kim and Connors, 2012). With regard to inhibitory transmission, the function of GABA_A_ receptors is affected by temperature, with increased temperature reducing the apparent affinity for GABA and increasing single GABA channel conductance (Jenkins et al., 1999; Perrais and Ropert, 1999; Millingen et al., 2008). The impact of raised temperature on inhibitory synaptic transmission is manifest as an increased frequency of synaptic currents, correlated with increased firing of inhibitory interneurons (Perrais and Ropert, 1999; Kim and Connors, 2012). Elevated temperature also results in larger IPSCs with faster kinetics (Otis and Mody, 1992; Perrais and Ropert, 1999).

Tonic inhibition is dependent upon various factors, including GABA release and uptake, as well as the activation properties of the underlying extrasynaptic receptors—thus, the overall effect of temperature on tonic GABA_A_ transmission will reflect a convolution of temperature effects on multiple processes. For example, uptake of GABA is more efficient at physiological temperatures than at room temperature (Mitchell and Silver, 2000). It has been demonstrated that enhanced uptake at physiological temperature constrains the spillover of GABA from synapses and prevents the activation of perisynaptic δ subunit-containing GABA_A_ receptors in DGGCs (Wei et al., 2003). It is also of note that extrasynaptic GABA_A_ receptors are subject to functional modulation by phosphorylation and the actions of kinases/phosphatases may be temperature dependent (Machu et al., 2006; Abramian et al., 2010; Tang et al., 2010; Saliba et al., 2012; Bright and Smart, 2013). We have recently discovered that the overall effect of raising the recording temperature from room (21–23°C) to physiological levels (34–36°C) is to cause a 2-fold increase in the tonic conductance in DGGCs (Bright and Smart, 2013). More work is necessary to establish whether tonic currents in other brain areas are also elevated with increased temperature.

### Composition of recording solution

The basic composition of the aCSF used to bathe brain slices during recording is generally similar between different laboratories. However, differences arise in the additional ingredients that are sometimes added and these can play an important role in setting the amplitude of the tonic inhibition. The most obvious example of this is the addition of GABA to the aCSF. Tonic inhibition clearly relies upon the availability of an ambient GABA concentration sufficient to activate extrasynaptic GABA_A_ receptors, and whilst in many brain areas tonic inhibition can be recorded without supplementary GABA, in many studies, a low concentration of GABA is added to the aCSF. There is some discussion as to whether tonic GABA currents recorded under conditions of added GABA are more or less “physiological” than currents recorded in the absence of extra GABA (Glykys and Mody, 2007a). Indeed, it has been suggested that if 5 μM GABA is added to the aCSF, active GABA uptake within the slice may significantly reduce the ambient concentration to levels close to those measured *in vivo* (0.2–2.5 μM) and that this might provide a way of standardizing tonic GABA measurements (Glykys and Mody, 2006, 2007a).

However, there are some potential confounds to the approach of adding GABA to the aCSF, the most obvious being that it is unclear what the “physiologically-correct” ambient GABA level within the slice “should be.” Measurements of extracellular ambient GABA concentrations *in vivo* cover a large range (30 nM–2.5 μM; (Lerma et al., 1986; Tossman et al., 1986; Ding et al., 1998; Bianchi et al., 2003; Xi et al., 2003; de Groote and Linthorst, 2007) and vary according to brain region and behavioral state. For example, GABA levels in the pontine reticular formation and the thalamus are dependent on arousal state, showing variation between wakefulness, rapid-eye-movement (REM) and non-REM sleep (Kekesi et al., 1997; Vanini et al., 2011). Interestingly, in the ventral hippocampus, GABA levels rise to around 800 nM during active exploration (de Groote and Linthorst, 2007).

Microdialysis is the most widespread method used to measure concentrations of various neurotransmitters, including GABA, in the living brain. However, there is some debate as to the origin of the GABA (and glutamate) that is detected (Timmerman and Westerink, 1997; Del Arco et al., 2003; van der Zeyden et al., 2008). Given the questions surrounding the origin of the GABA, the conditions used for measuring dialysate GABA have recently been critically re-examined (van der Zeyden et al., 2008). It was shown that the most widely used chromatographic method for assay of GABA is extremely sensitive to small changes in analytical conditions, particularly pH, and that unless conditions are optimized, measured GABA levels may be artifactually elevated.

Using optimized conditions, these investigators report basal GABA levels of only 2–4 nM in various brain regions, which are significantly lower than in other studies. Since microdialysis probes are also known to create a disrupted zone of tissue that may well influence local neurotransmitter levels, it was suggested that the development of microsensors to assay GABA may be necessary to fully define extracellular GABA concentrations within the brain. This may be compounded by complex tissue topography in the CNS and the potential for GABA concentrations to vary between tissue compartments. Adding GABA may also cause problems since increased GABA levels may recruit additional populations of receptors to contribute to tonic inhibition (Scimemi et al., 2005) as well as desensitizing high affinity δ-GABA_A_ receptors (Mortensen et al., 2010; Bright et al., 2011). There is also a need to consider how the ambient GABA concentration may influence drug modulation of these receptors (Houston et al., 2012).

Other additions to the aCSF may be made to enhance slice viability, particularly during the slicing process. Common examples of these include sodium pyruvate, ascorbic acid and indomethacin. However, it should be noted that some of these supplementary compounds may also cause variation in the measured tonic current. In particular, it is well established that GABA_A_ receptors are subject to modulation by redox reagents (Pan et al., 1995; Amato et al., 1999). A recent study has demonstrated that the tonic current mediated by GABA_C_ receptors in retinal bipolar cells is significantly enhanced by the endogenous reducing agent, ascorbic acid (Calero et al., 2011). Given that receptors lacking a γ2-subunit are more sensitive to the actions of redox reagents (Amato et al., 1999), extrasynaptic δ-GABA_A_ receptors may also be significantly modulated by redox compounds. Indeed, it may be that recordings within brain slices underestimate tonic current amplitudes due to wash-out of endogenous reducing agents such as ascorbic acid (Hajos and Mody, 2009).

## Measurement and analysis of tonic inhibition

The standard method to reveal the presence of a tonic GABA current in a whole-cell recording is to apply a saturating concentration of a specific GABA_A_ receptor antagonist, usually bicuculline, gabazine (SR99531) or picrotoxin (PTX). As well as blocking IPSCs, this treatment will reveal a tonic inhibition by causing a shift in the holding current (outward if E_Cl_ is set to be close to 0 mV as is often the case), and a reduction in the baseline current variance, consistent with a decrease in the number of open GABA_A_ channels.

### Pharmacological blockade of GABA_A_ receptors

Care should be taken both in the choice of antagonist and its concentration. For instance, bicuculline can either be used as a pure base or as one of a number of charged quaternary salts (methoiodide, methochloride or methobromide). The quaternary salts are used experimentally due to their greater water solubility compared with the base. However, these bicuculline salts are not specific blockers of GABA_A_ receptors since they will also block some Ca^2+^-activated K^+^ channels at similar concentrations (Seutin and Johnson, 1999).

It is also important to note that the different antagonists exhibit different mechanisms of action. Picrotoxin acts as a potent mixed GABA_A_ receptor antagonist (Smart and Constanti, 1986). The conventional view has been that the action of picrotoxin results from binding to a site within the ion channel, which results in a steric hindrance of ion flow (Takeuchi and Takeuchi, 1969; Inoue and Akaike, 1988; Sedelnikova et al., 2006; Erkkila et al., 2008). However, various studies have shown features of picrotoxin inhibition that are inconsistent with a simple channel-block mechanism and it has therefore been postulated that picrotoxin may also allosterically stabilize one or more non-conducting (i.e., closed or desensitized) states of the channel (Smart and Constanti, 1986; Newland and Cull-Candy, 1992; Krishek et al., 1996; Korshoej et al., 2010). In contrast, bicuculline and gabazine are both competitive antagonists that displace GABA from the agonist binding site and therefore prevent receptor activation (Akaike et al., 1987). Additionally, both these drugs act as negative allosteric inhibitors of channel opening, inhibiting activation of GABA_A_ receptors by anaesthetic agents, with bicuculline being more effective in this respect (Ueno et al., 1997). This greater inverse agonist activity is likely to explain the ability of bicuculline, but not gabazine, to inhibit spontaneous GABA-independent channel opening in hippocampal pyramidal cells and DGGCs (Bai et al., 2001; Mtchedlishvili and Kapur, 2006; McCartney et al., 2007; Wlodarczyk et al., 2013). Conversely, the lower inverse agonist activity of gabazine may allow it to selectively block synaptic GABA_A_ receptors at lower concentrations without affecting the extrasynaptic receptors mediating tonic inhibition (Stell and Mody, 2002; Park et al., 2007; Yamada et al., 2007). Caution should also be exercised when using high concentrations of gabazine (>100 μM) since it appears to show some agonist activity (Wlodarczyk et al., 2013).

Some studies have used subtype-selective blockers of GABA_A_ receptors to identify specific components of the tonic current. The first identified example of a subtype-selective antagonist was furosemide which blocks α6-containing receptors with high affinity (Korpi et al., 1995). Accordingly, concentrations of up to ~100 μM furosemide that are ineffective at other GABA_A_ receptors or importantly, at K^+^Cl^−^ cotransporters (Hamann et al., 2002) produce a significant blockade of the α6δ-mediated tonic current in cerebellar granule cells (Hamann et al., 2002; Wall, 2002; Bright et al., 2011). At higher concentrations (100–1000 μM), furosemide blocks α4-containing receptors (Wafford et al., 1996) and has been used to inhibit the tonic conductance mediated by these receptors in hippocampal neurons (Mangan et al., 2005; Mtchedlishvili and Kapur, 2006).

Subtype selective inhibition is also provided by the benzodiazepine-site inverse agonist, L-655,708, which blocks α5-containing receptors with at least 50-fold selectivity over receptors containing α1/2/3/6 subunits (Quirk et al., 1996; Casula et al., 2001). This compound has been shown to partially-block tonic currents mediated by α5-receptors in pyramidal cells of both the hippocampus and cortex (Caraiscos et al., 2004; Scimemi et al., 2005; Yamada et al., 2007; Glykys et al., 2008). It should be noted that, whilst radiolabelling with L-655,708 demonstrates that this compound binds with high affinity (*K*_*D*_ ~ 2.5 nM, (Quirk et al., 1996) which correlates with a high potency for block of recombinant α5β3γ2 receptors (IC_50_ = 1.1 nM, Atack et al., 2006), the maximum block achievable is limited (~17%, Atack et al., 2006). Therefore, L-655,708 is a very weak partial inverse agonist and can only achieve partial blockade of α5-mediated tonic currents.

The cation Zn^2+^ is a useful pharmacological tool to separate components of tonic inhibition since it blocks αβ and αβδ receptors with significantly higher potency (respective IC_50_s 88 nM and 6–16 μM) than receptors containing the γ2 subunit (IC_50_ 300 μM; (Krishek et al., 1998; Hosie et al., 2003). Recordings from cultured hippocampal pyramidal neurons revealed that a component of the tonic current was highly sensitive to inhibition by Zn^2+^, suggesting the potential contribution (~10%) of αβ GABA_A_ receptor isoforms (Mortensen and Smart, 2006). More generally, application of Zn^2+^ has been used to detect the involvement of non-γ2 containing receptors in the generation of tonic currents (Jia et al., 2005; Nani et al., 2013).

Given the sensitivity to Zn^2+^, it is perhaps not surprising that recently, Cu^2+^, which shares many characteristics with Zn^2+^ in terms of coordination and is known to inhibit invertebrate GABA receptors (Irving and Williams, 1953; Smart and Constanti, 1982), has been demonstrated to inhibit GABA_A_ receptors underlying tonic inhibition with high selectivity over synaptic GABA_A_ receptors (McGee et al., 2013). The trivalent cation, La^3+^, is also a selective blocker at certain extrasynaptic GABA_A_ receptors, showing a potent inhibition at α4/6βδ receptors, a weaker inhibition at α4/6βγ2 and α4β receptors, and a potentiation of GABA at α1βδ/γ2 receptors (Im et al., 1992; Saxena et al., 1997; Zhu et al., 1998; Brown et al., 2002; Storustovu and Ebert, 2006). However, La^3+^ has only been used infrequently in studies of tonic conductances (Shen et al., 2005), probably due to its low efficacy of inhibition (Storustovu and Ebert, 2006).

### Selective activation/modulation of extrasynaptic GABA_A_ receptors

Many studies demonstrate relatively small tonic GABA_A_ current amplitudes under basal conditions in brain slice preparations. It is therefore often advantageous to boost the tonic current by addition of either a selective GABA_A_ agonist or a positive allosteric modulator. The GABA_A_ agonist, THIP (4,5,6,7-tetrahydoisoxazolo[5,4-c]pyridin-3(2*H*)-one) has been extensively characterized as a “super-agonist” at both αβ and αβδ receptors (Adkins et al., 2001; Brown et al., 2002; Storustovu and Ebert, 2006; Mortensen et al., 2010), whilst displaying only partial agonist activity at synaptic αβγ receptors (Ebert et al., 1994; Mortensen et al., 2004). THIP therefore provides a useful selective agonist for activation of the tonic current and has been widely used in this context (e.g., Cope et al., 2005; Maguire et al., 2005; Drasbek and Jensen, 2006).

Selective positive allosteric modulation of extrasynaptic GABA_A_ receptors may also provide a useful tool for detection and characterization of the tonic current. Endogenous neurosteroids act as potent positive allosteric modulators at all major GABA_A_ receptor subtypes (Belelli et al., 2002; Belelli and Lambert, 2005) by binding to a defined site on the receptor α subunit (Hosie et al., 2006, 2009). Studies in recombinant systems suggest that receptors containing the δ subunit are more sensitive to neurosteroid-induced potentiation than those containing a γ subunit (Belelli et al., 2002; Brown et al., 2002; Wohlfarth et al., 2002). Consistent with this, in some brain areas the tonic GABA_A_ current appears to be more sensitive to neurosteroid modulation than synaptic GABA_A_ currents (e.g., Stell et al., 2003; Maguire et al., 2005; Bright and Smart, 2013). However, caution should be exercised in using neurosteroids since in some cases, synaptic inhibition may be equally, or more sensitive, than the tonic current (Porcello et al., 2003; Cope et al., 2005). Diversity in neurosteroid modulation may arise from variations in ambient GABA level, differences in receptor phosphorylation and the direction of Cl^−^ flux (reviewed in Herd et al., 2007).

More recently, a novel compound, DS2, has been identified as a selective modulator of δ-containing GABA_A_Rs (Wafford et al., 2009; Jensen et al., 2013). This compound acts via a new binding site on α4/6βδ receptors to enhance the efficacy of GABA, whilst having little action on αβ or αβγ receptors (Jensen et al., 2013). As expected from the recombinant data, DS2 selectively potentiates the tonic current in thalamic relay neurons with no effect on IPSCs (Wafford et al., 2009; Jensen et al., 2013). Thus, DS2 may represent a more useful tool than neurosteroids for selective modulation of extrasynaptic GABA_A_ receptor activity.

### Characterization of tonic current *in vivo*

Most studies of tonic inhibition have been carried out *in vitro* in brain slice preparations. However, it remains an important question to determine how the tonic GABA current impacts upon neuronal excitability *in vivo*. To date, only a few studies have recorded tonic currents within the living brain. In cerebellar granule cells, a tonic current is manifest upon application of a high concentration (0.5 mM) of the specific antagonist, gabazine (Chadderton et al., 2004; Duguid et al., 2012). Conversely, the tonic current can be selectively activated by application of THIP (Duguid et al., 2012). The tonic inhibition generated by extrasynaptic GABA_A_ receptor activation appears to be carefully tuned to provide high fidelity transmission of sensory information since either enhancement or blockade leads to a reduction in the signal-to-noise ratio (Duguid et al., 2012). A recent study has also characterized tonic inhibition in granule cells of the olfactory bulb *in vivo* (Labarrera et al., 2013). In these cells, the tonic current was blocked by application of gabazine or furosemide, with, in the case of the latter antagonist, little effect on inhibitory synaptic currents. The selective block of the tonic current with furosemide was used to show that tonic inhibition dominates over the phasic inhibition stimulated by odor presentation and thus provides a key regulator of neuronal excitability. Recording of tonic currents *in vivo* is complicated by the necessity of using high antagonist concentrations to ensure penetration to the site of action and by the fact that drug wash-out is not possible.

### Measurement of tonic current

**Figure 1:**
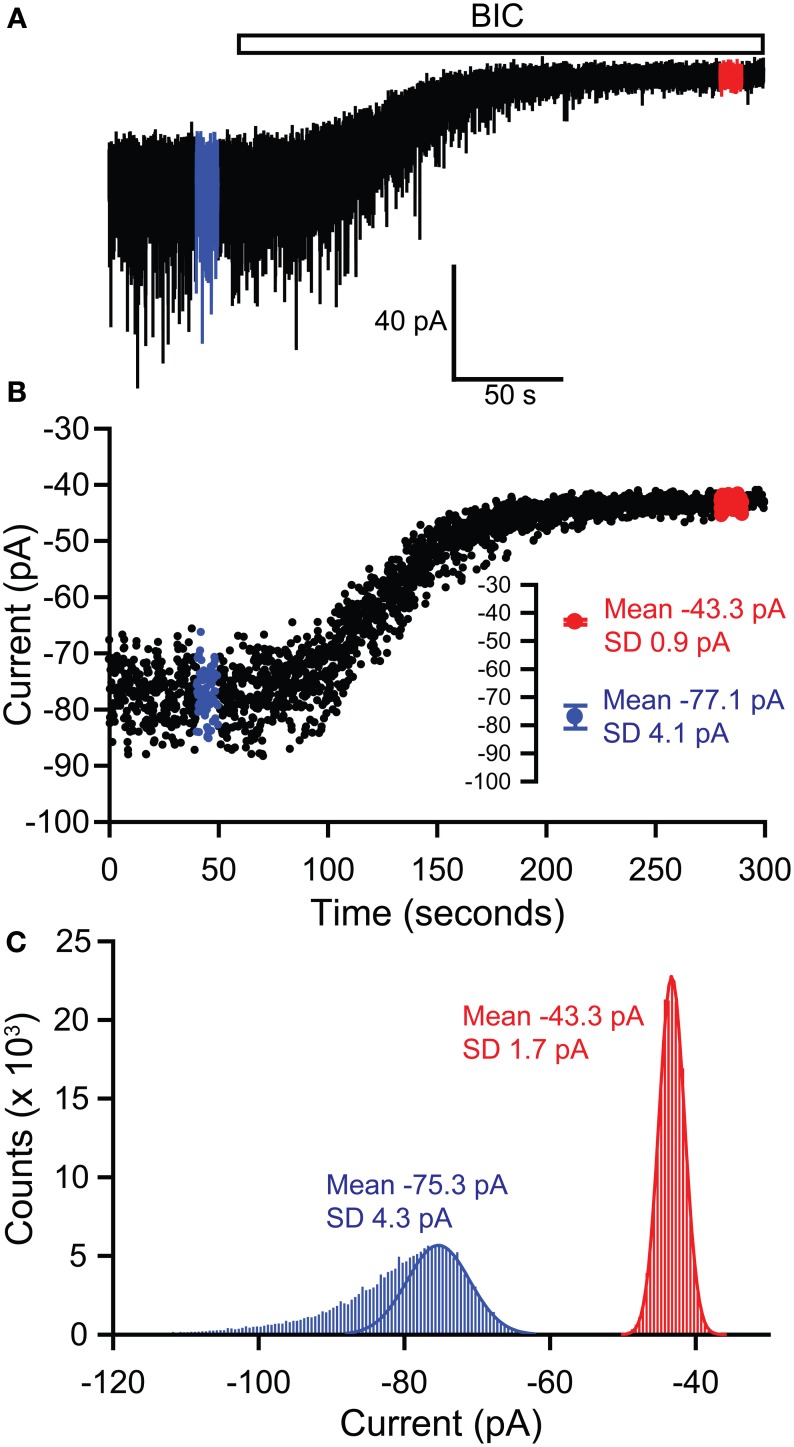
**Methods of measuring tonic GABA current shifts. (A)** Example current trace recorded from a thalamic relay neuron illustrating block of sIPSCs by application of bicuculline (BIC) and the simultaneous block of a tonic GABA current, revealed by the outward shift in the holding current. Epochs used to define the holding current under control and bicuculline conditions are illustrated in blue and red respectively. **(B)** The holding current is sampled by averaging over 5 ms at 100 ms intervals. Any of these baseline points falling on the decay of a sIPSC are omitted, before plotting against time to give the current-time plot shown. Values for the current in control and bicuculline are then calculated by averaging over the 10 s long epochs shown in blue and red respectively—these mean values are shown in the inset. Using this method, the tonic current is 77.1 − 43.3 = 33.8 pA. **(C)** The tonic current can also be defined by generating all-points histograms for the control and bicuculline epochs and then fitting Gaussian curves to the positive side of these histograms. The mean values from the Gaussian fits are then used to define the currents in control and bicuculline. This method thus gives a tonic current of 75.3 − 43.3 = 32 pA.

The magnitude of the tonic current mediated by extrasynaptic GABA_A_ receptors is typically revealed by measuring the change in the holding current evoked by applying a GABA antagonist. Since most neuronal recordings will also include inhibitory synaptic currents, it is important to exclude these from the tonic current measurement. There are two simple methods that are generally used to measure the holding current shift (Figure 1). Firstly, the baseline current can be digitally sampled by averaging over short epochs (1–10 ms) at regular intervals (100–1000 ms). Any baseline points falling on the decay of an IPSC are simply omitted before averaging the remainder to obtain “clean” measures of the holding current before and after antagonist application.

Alternatively, the baseline current can be analyzed by generating an all-points histogram and fitting a Gaussian distribution to the positive side of this histogram, i.e., the side that is uncontaminated by the negative-going IPSCs which will skew the distribution (Figure 1; (Nusser and Mody, 2002). Then, the means of the fitted Gaussians are used to define the holding currents before and after antagonist application. In practice, both of these methods for calculating baseline shifts tend to produce nearly identical results. The second method has been refined to allow the simultaneous measurement of tonic and phasic inhibitory currents and to establish any temporal correlation between the two forms of inhibition (Glykys and Mody, 2007b). Tonic currents are often normalized to the cell capacitance (pA/pF) to allow comparison between cells of different sizes and may also be expressed as conductances (pS/pF) to allow comparison with other active channel populations.

### Use of current noise

Under conditions where the tonic current amplitude is small, it may be useful to use changes in the current noise as a way of monitoring changes in the activity of tonically open GABA_A_ receptors (Mtchedlishvili and Kapur, 2006; Glykys and Mody, 2007a). The current noise may be quantified as either the variance (σ^2^) or the RMS noise (the square root of the variance, equivalent to the standard deviation of the signal), defined using the following equations.

$$\sigma^{2}=\frac{1}{n}\sum_{x\;=\;1}^{n}{\left(I\left(x\right)-I\right)}^{2}$$

where *n* is the number of samples, *I*(*x*) is the current measured for the *x*th sample and *I* is the mean current defined by:

$$I=\frac{1}{n}\sum_{x\;=\;1}^{n}I(x)$$

If we assume that the channels function independently from each other and are either open or closed (i.e., only two states), then the mean current and variance can also be expressed using the channel open probability, *P*, and the single channel current, *i*, as:

(1) I=NPi

(2) $$\sigma^{2}=Ni^{2}P\text{​}\left(1-P\right)$$

These equations (see (Hille, 1992) for derivations) can then be combined to give:

(3) $$\sigma^{2}\left(I\right)=iI-\frac{I^{2}}{N}$$

Equation (3) relates the current *I* to the variance σ^2^. This is a parabolic equation with two roots at *I* = 0 and *I* = *iN*. Hence, there will be no noise when all of the channels are either fully shut (*P* = 0) or fully open (*P* = 1).

Equation (3) provides the basis for the stationary and non-stationary noise analysis techniques which have been widely used to obtain estimates of *i, N*, and *P* for both voltage and ligand gated ion channels (Sigworth, 1980; Traynelis and Jaramillo, 1998; Lingle, 2006; Hartveit and Veruki, 2007). With regard to inhibitory transmission, non-stationary noise analysis has been used in many studies to estimate the underlying single channel conductance and open probability for the synaptic GABA_A_ receptors that are responsible for fast phasic inhibition (for example, De Koninck and Mody, 1994; Nusser et al., 1997; Houston et al., 2008; Bright et al., 2011). However, noise analysis techniques have only infrequently been applied to extrasynaptic GABA_A_ receptor activation (Kaneda et al., 1995; Bai et al., 2001; Naylor et al., 2005). We wanted to assess the conditions under which changes to membrane current noise would be a more advantageous measure compared with changes in tonic current for assessing variations in tonic inhibition.

To do this, we have used simulated channel noise generated by the ion channel noise simulation module of WinEDR software (V3.3.8, John Dempster, University of Strathclyde, UK). This uses a simple 2-state model to produce the random fluctuations in current associated with stochastic opening and closing of a population of ion channels. Simulations were configured to mimic our typical conditions for recording tonic currents within brain slice preparations—therefore, we used a sampling interval of 0.05 ms (equivalent to a sampling frequency of 20 kHz) and a background noise variance of 2 pA (measured from room temperature recordings in DGGCs).

Simulation parameters associated with the ion channel population are the single channel current, *i*, the number of channels, *N*, the probability of channel opening, *P* and the open time constant, τ_open_. Setting of these parameters was guided by values obtained from an earlier study in our lab of agonist-activated single-channel currents in outside-out patches from HEK293 cells expressing recombinant α4β3δ GABA_A_ receptors (Mortensen et al., 2010). Thus, the single channel current i is ~2 pA (equivalent to a conductance of ~30 pS), the maximum open probability *P* is ~0.1 (for activation by GABA) and the mean open time constant τ_open_ is ~1 ms. The number of channels *N* was arbitrarily set to be 100.

Tonic inhibition may be modulated by changes in any of the three underlying parameters: single channel current, number of channels and the open probability. Thus, we will consider the impact of changes to these parameters on simulated tonic current and noise.

#### Changes in open probability

**Figure 2:**
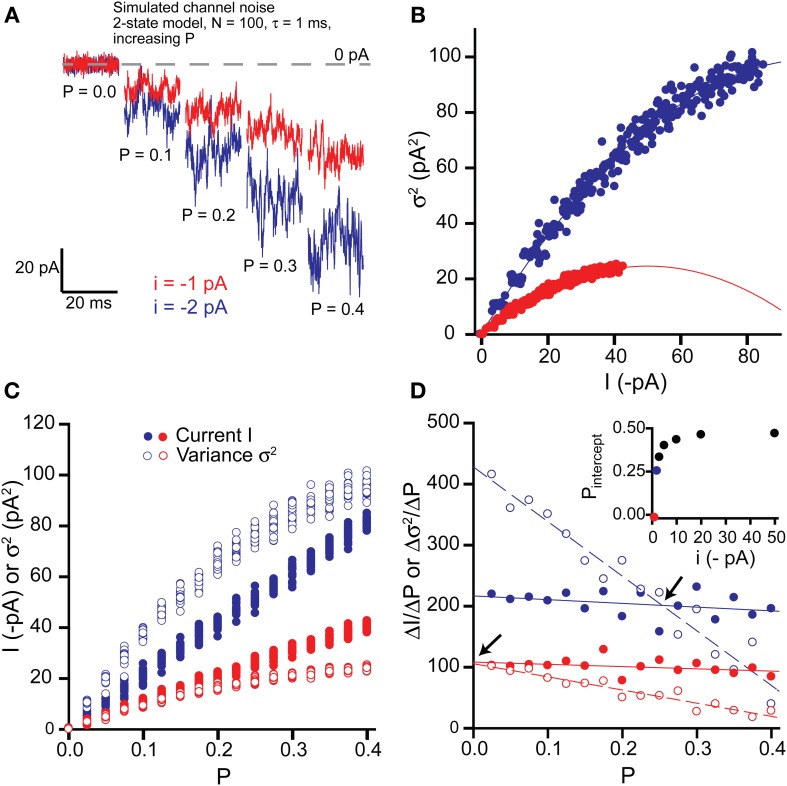
**Effects of changing open probability *P* on simulated tonic current and noise. (A)** Simulated “noisy” currents generated by using a simple 2-state model in WinEDR. Simulation parameters used for the ion channels were number of channels *N* = 100 and open time constant τ = 1 ms with a background noise of 2 pA. Open probability *P* was increased from 0 to 0.4 in 0.025 increments of which only selected values are shown. Example currents are shown for single channel currents *i* = −1 and −2 pA. **(B)** Plots of the mean current I against variance σ^2^ illustrate the expected parabolic relationship—data points are fitted well with equation (3). **(C)** Plots of mean current I and variance σ^2^ against open probability *P* for the two selected single channel currents. **(D)** The slopes of the *I*-*P* and σ^2^-*P* plots were calculated (ΔI/Δ*P* and Δσ^2^/Δ*P*) and plotted against *P*. Linear regression was performed on the slope plots (solid lines for Δ*I*/Δ*P* and dashed lines for Δσ^2^/Δ*P*) and the points of intersection between these lines are indicated by the arrows. The *P* value at these points of intersection, *P*_intercept_ is plotted against the single channel current (inset).

To assess the impact of changes in channel open probability, we simulated 20 s long stretches of channel opening for increasing values of *P* from 0 to 0.4 in 0.025 increments (Figure 2). Baseline current was sampled by averaging over 5 ms epochs every 100 ms and then the mean current was calculated by averaging over 1 s intervals (i.e., 10 baseline points per mean value, 20 mean current points per 20 s long activation). Baseline variance was calculated over 100 ms epochs, before averaging to give a mean variance for each 1 s interval.

Figure 2A shows current segments generated using increasing values of *P* up to 0.4 for single channels with currents of −1 and −2 pA. The maximum open probability used for these simulations is about 4-fold higher than expected for activation of δ-GABA_A_Rs by GABA (Mortensen et al., 2010). Plotting variance vs. mean current plots revealed the expected parabolic curves and these were well fitted by equation (3) (Figure 2B). Estimates of *N* and *i* obtained from the parabolic fits correlated well with the expected theoretical values (*i* = −1 pA: *N* = 101.0, *i* = −0.99 pA; *i* = −2 pA: *N* = 101.0, *i* = −1.98 pA).

To determine whether the variance σ^2^ is a more sensitive indicator of changes in the open probability *P* than the current *I*, we plotted both σ^2^ and *I* against *P* (Figure 2C). We then calculated the slopes for the *I-P* and σ^2^-*P* plots (Δ*I*/Δ*P* and Δσ^2^/Δ*P*) and plotted these against *P* (Figure 2D). For a given change in *P*, the absolute value of the variance will change more than the absolute value of the current if Δσ^2^/Δ*P* is greater than Δ*I*/Δ*P*. For example, for the case of a single channel current *i* = −1 pA, Δσ^2^/Δ*P* is always less than Δ*I*/Δ*P* and therefore, the current will be a more sensitive indicator of changes in *P*. We have quantified this relationship for various values of i (−1, −2, −3, −5, −10, −20, −50 pA) by performing linear regression on the slope plots and finding the *P* value where each pair of straight lines cross (*P*_intercept_). Thus, we can see that for increasing values of *i*, the range of *P* values over which Δσ^2^/Δ*P* is greater than Δ*I*/Δ*P* increases and *P*_intercept_ tends toward 0.5 (inset, Figure 2D). However, for the low values of *i* expected for physiological activation of δ-GABA_A_Rs (up to about −2 pA), whether the variance is a more sensitive indicator of changes in open probability will depend upon both *P* and *i*. Thus, for single channel current of −1 pA or less, the current will always be a better indicator of changes in *P*, whilst for −1 pA < *i* < −2 pA, the variance will be more sensitive when *P* is low (less than ~0.25 for *i* = −2 pA).

#### Changes in number of receptors

**Figure 3:**
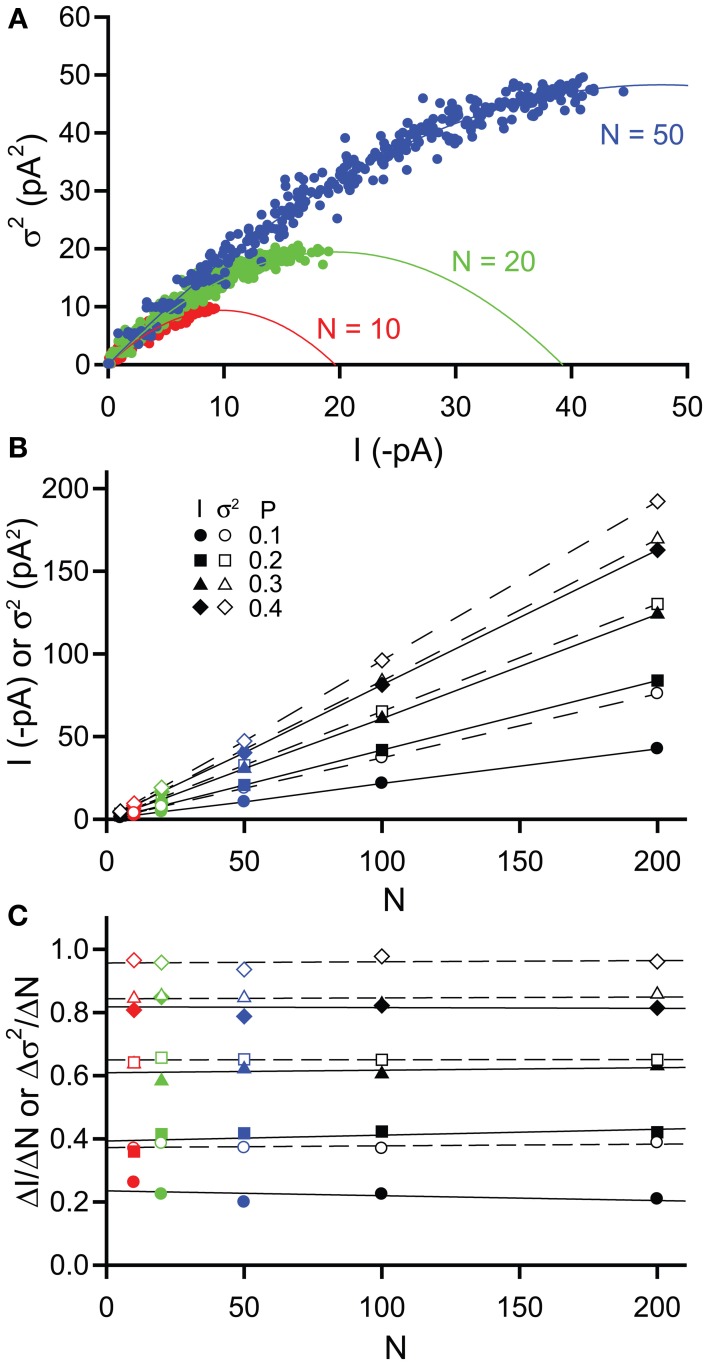
**Effects of changing channel number *N* on simulated tonic current and noise**. Simulated currents were generated in WinEDR to investigate the impact of changing channel number *N* on current and variance. Open probability *P* was increased from 0 to 0.4 in 0.025 increments **(A)** Plots of the mean current I against variance σ^2^ for *N* = 10, 20, & 50 illustrate the expected parabolic relationship—data points are fitted well with equation (3). **(B)** Plots of mean current *I* (filled symbols, solid lines) and variance σ^2^(open symbols, dashed lines) against channel number *N* for *P* values of 0.1, 0.2, 0.3, & 0.4. **(C)** The slopes of the *I*-*N* and σ^2^-*N* plots were calculated (Δ*I*/Δ*N*, filled symbols and Δσ^2^/Δ*N*, open symbols) and plotted against *N*. Linear regression was performed on the slope plots (solid lines for Δ*I*/Δ*N* and dashed lines for Δσ^2^/Δ*N*). The slopes are constant for both current and variance but for a given *P* value, Δσ^2^/Δ*N* is always greater than Δ*I*/Δ*N*.

The impact of changing the number of channels (*N*) was investigated by again simulating stretches of channel opening with increasing *P* (0–0.4 in 0.025 increments as before with a single channel current *i* = −2 pA) but this time we also varied the value of *N* (5, 10, 20, 50, 100, 200). Figure 3A shows example current-variance plots generated for values of *N* = 10, 20, & 50, that are again well fitted by the expected parabolic relationship [equation (3)]. Plotting current and variance against *N* illustrates that both increase linearly and that for values of *P* up to 0.4, the variance is always greater than the current (Figure 3B). Inspection of equations (1) and (2) shows that this linearity is expected since both current and variance are proportional to *N*. Hence, for a given change in *N*, both current and variance will change by the same relative amount e.g., doubling *N* will causes a doubling of both current and variance. However, plotting the slopes of the current and variance with respect to *N* (Δ*I*/Δ*N* and Δσ^2^/ΔN) reveals that both slopes are constants for a given *P* value (as expected) and that the slope of the variance is always greater than that of the current (Figure 3C). Hence, for a given change in *N*, the absolute value of the variance will change more than the absolute value of the current. This simple analysis would suggest that over the range of open probabilities expected for extrasynaptic δ-GABA_A_R activation by GABA (0–0.1; Mortensen et al., 2010), using changes in variance might be a more sensitive method for detecting changes in receptor number *N*.

#### Changes in single channel current

Finally, we examined the effect of changing the single channel current *i*. These simulations were also performed for increasing values of *P* (0–0.4 in 0.025 increments) with a fixed number of channels *N* = 100, whilst we varied the value of *i*. Compared with the simulations performed in section 2.3.1, we used lower values of *i* (−0.25, 0.5, −1.0, −1.5, −2.0, and −5.0 pA). Under physiological conditions when the intracellular Cl^−^ ion concentration is typically low, the driving force for Cl^−^ ion flux will be small, therefore leading to a reduced single channel current for GABA_A_ receptors (e.g., for a driving force of 10 mV with a single channel conductance of 30 pS, *i* = 0.3 pA).

**Figure 4:**
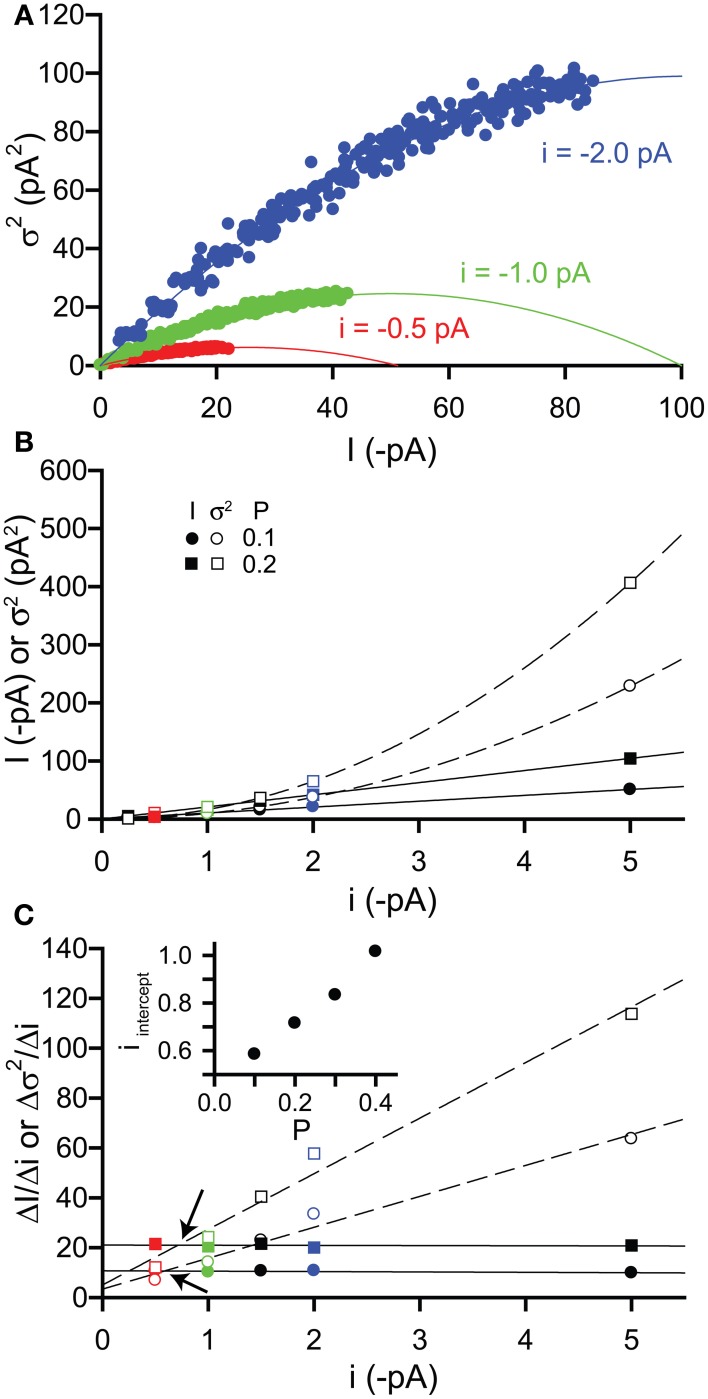
**Effects of changing single channel current i on simulated tonic current and noise**. Simulated currents were generated in WinEDR to investigate the impact of changing single channel current i on current and variance. Open probability *P* was increased from 0 to 0.4 in 0.025 increments with a fixed number of channels *N* = 100. **(A)** Plots of the mean current I against variance σ^2^ for *i* = −0.5, −1, & −2 pA illustrate the expected parabolic relationship—data points are fitted well with equation (3). **(B)** Plots of mean current *I* (filled symbols, solid lines are linear fits to data points) and variance σ^2^ (open symbols, dashed lines represent fitting of a quadratic equation to the data points) against single channel current *i* for *P* values of 0.1 & 0.2. **(C)** The slopes of the *I*-*i* and σ^2^-*i* plots were calculated (Δ*I*/Δ*i*, filled symbols and Δσ^2^/Δ*i*, open symbols) and plotted against *i*. Linear regression was performed on the slope plots (solid lines for Δ*I*/Δ*i* and dashed lines for Δσ^2^/Δ*i*) and the points of intersection between these lines are indicated by the arrows. The *i* value at these points of intersection, *i*_intercept_ is plotted against the open probability *P* (inset).

Figure 4A shows example current-variance plots generated for *i* = −0.5, −1, & −2 pA that are again well fitted by the expected parabolic relationship [equation (3)]. If we plot both current and variance against *i*, then we can see that current increases linearly with *i*, as expected from equation (1), whilst the variance increases with the square of *i*, as expected from equation (2) (Figure 4B). To establish when the variance is a more sensitive indicator of changes in *i* than the current, we have plotted the slopes of both (Δ*I*/Δ*i*, Δσ^2^/Δ*i*) with respect to *i* (Figure 4C). As expected, for a given value of *P*, the slope of the current is constant whilst that of the variance increases linearly. We have quantified when Δσ^2^/Δ*i* is greater than Δ*I*/Δ*i* by performing linear regression on the slope points and finding the value of i at the point of intersection between these two lines (*i*_*intercept*_). Thus, when *i* is greater than *i*_intercept_ then the variance should be a more sensitive measure of changes in *i*. From Figure 4C, we can see that *i*_intercept_ increases with open probability *P*. For the low values of *P* expected for the activation of δ-GABA_A_Rs by GABA (*P* < 0.1), the variance will change more than the current when *i* is greater than about 0.6 pA. For a 30 pS single channel conductance, this is equivalent to a driving force for Cl^−^ ions of 20 mV. This indicates that under physiological conditions when the extrasynaptic GABA_A_ receptor conductance is more shunting than hyperpolarizing, changes in macroscopic current should be a more sensitive indicator.

#### Summary—when to use current or variance

Using a simple analysis of simulated current noise, we have investigated the conditions under which analysis of the current variance might prove a more sensitive way of determining changes in extrasynaptic receptor activity than monitoring changes in current. Changes in tonic inhibition might result from modulation of receptor open probability *P*, changes in receptor number *N* and/or modulation of single channel current *i*. Of these, perhaps changes in *P* are most likely, since this will occur when the ambient GABA concentration varies or when activation properties of extrasynaptic receptors are altered (e.g., by phosphorylation). Our analysis would suggest that for the low values of *i* that are expected when cells are not chloride-loaded, the macroscopic current is a better indicator of changes in *P*. For higher values of i (−1 pA < *i* < −2 pA), the variance will be a more sensitive monitor, if *P* remains relatively low (less than ~0.25). Hence, under the usual experimental conditions for recording tonic inhibition (i.e., with high intracellular Cl^−^ concentration), it might well be better to look for changes in variance.

Changes in tonic inhibition resulting from altered receptor number *N* might arise if there is an alteration in receptor trafficking. At the moment, very little is known about what controls the surface expression and targeting of extrasynaptic GABA_A_ receptors compared with their synaptic counterparts (Moss and Smart, 2001; Arancibia-Carcamo and Kittler, 2009; Luscher et al., 2011). We have recently shown that surface expression of δ-GABA_A_ receptors is reduced by activation of protein kinase C (Bright and Smart, 2013) and it would be surprising if other signaling cascades do not impinge upon extrasynaptic receptor trafficking. Our analysis would suggest that changes in receptor number *N* lead to a greater change in the variance than the current. Hence, over the range of open probabilities that we have examined (up to *P* = 0.4), the current variance is a more sensitive indicator of changes in *N*.

Although it is generally accepted that the underlying single channel conductance of GABA_A_ receptors is not affected by modulators (though c.f. Eghbali et al., 1997), the single channel current will be affected by changes in the ionic driving force. For extrasynaptic GABA_A_ receptors, changes in single channel current i will occur if the reversal potential for Cl^−^ ions is altered, under conditions of altered Cl^−^ homeostasis or if the neuronal membrane potential is changed. Our analysis would indicate that when the single channel current is small (i.e., when the tonic conductance is more shunting than hyperpolarizing), then changes in membrane current represent a more sensitive means of detecting changes in *i.* However, when the single channel current is greater, (for instance, when GABA_A_ receptor activity is recorded under conditions of symmetrical intracellular and extracellular Cl^−^ ion concentration), then the variance will be more sensitive to changes in *i*.

## Concluding remarks

Tonic GABA_A_ receptor-mediated conductances have been recorded under a multiplicity of different experimental conditions, many of which may impact upon the magnitude of the recorded conductance. It would not be possible to recommend one set of “ideal” conditions under which to record tonic inhibition, since experimental methods are determined by many other factors. However, experimenters should be aware of the potential contributions of various experimental conditions, including the age of the animal, how brain slices are stored and perfused, the recording temperature and the composition of the recording solution, in determining the amplitude of the tonic conductance.

Tonic currents are revealed by the application of a specific GABA_A_ receptor antagonist. Care should be taken with both the choice and concentration of antagonist used since some blockers may not be absolutely selective and different blockers may exhibit distinct pharmacological modes of action. The tonic current is measured by quantifying the current shift upon blockade of GABA_A_ receptors, taking care to exclude the contribution of synaptic currents. There are two established methods for achieving this (described in section Measurement of Tonic Current), both of which tend to produce nearly identical results.

The use of current noise may provide a complementary approach, both for establishing the presence of a tonic conductance and for monitoring changes in extrasynaptic receptor activity. Our analysis of simulated currents suggests that monitoring changes in current variance may provide a more sensitive way of detecting changes in tonic inhibition under specific conditions than simply measuring changes in current. In particular, it is noteworthy that for the low open probabilities expected for activation of δ-GABA_A_ receptors, changes in receptor number *N*, manifest upon altered trafficking, would be more easily detected from changes in the variance than the current. It should be noted that we have used a simple two-state model for generating current activations and that the resulting current-variance relationships are always parabolic. However, it has been shown for synaptic receptor activation that the shape of current-variance relationships can be transformed from parabolic to skewed, depending upon the temporal structure of underlying current fluctuations (Hartveit and Veruki, 2006). At the moment, it is not clear whether physiological extrasynaptic receptor activation generates a parabolic or skewed current-variance relationship. Nevertheless, given that the variance is a “free” signal and is easily measured, it may well represent a useful way of monitoring changes in tonic GABA_A_ receptor activity.

### Conflict of interest statement

The authors declare that the research was conducted in the absence of any commercial or financial relationships that could be construed as a potential conflict of interest.

## References

[B1] Abramian A. M. Comenencia-Ortiz E. Vithlani M. Tretter E. V. Sieghart W. Davies P. A. (2010). Protein kinase C phosphorylation regulates membrane insertion of GABA_A_ receptor subtypes that mediate tonic inhibition. J. Biol. Chem. 285, 41795–41805 10.1074/jbc.M110.149229 20940303 PMC3009907

[B2] Adkins C. E. Pillai G. V. Kerby J. Bonnert T. P. Haldon C. McKernan R. M. (2001). α4β3δ GABA_A_ receptors characterized by fluorescence resonance energy transfer-derived measurements of membrane potential. J. Biol. Chem. 276, 38934–38939 10.1074/jbc.M104318200 11495904

[B3] Akaike N. Yakushiji T. Tokutomi N. Carpenter D. O. (1987). Multiple mechanisms of antagonism of gamma-aminobutyric acid (GABA) responses. Cell Mol. Neurobiol. 7, 97–103 10.1007/BF00734993 3594520 PMC11567227

[B4] Amato A. Connolly C. N. Moss S. J. Smart T. G. (1999). Modulation of neuronal and recombinant GABA_A_ receptors by redox reagents. J. Physiol. 517, 35–50 10.1111/j.1469-7793.1999.0035z.x 10226147 PMC2269321

[B5] Arancibia-Carcamo I. L. Kittler J. T. (2009). Regulation of GABA_A_ receptor membrane trafficking and synaptic localization. Pharmacol. Ther. 123, 17–31 10.1016/j.pharmthera.2009.03.012 19374920

[B6] Atack J. R. Bayley P. J. Seabrook G. R. Wafford K. A. McKernan R. M. Dawson G. R. (2006). L-655,708 enhances cognition in rats but is not proconvulsant at a dose selective for α5-containing GABA_A_ receptors. Neuropharmacology 51, 1023–1029 10.1016/j.neuropharm.2006.04.018 17046030

[B7] Bai D. Zhu G. Pennefather P. Jackson M. F. MacDonald J. F. Orser B. A. (2001). Distinct functional and pharmacological properties of tonic and quantal inhibitory postsynaptic currents mediated by gamma-aminobutyric acid_*A*_ receptors in hippocampal neurons. Mol. Pharmacol. 59, 814–824 10.1124/mol.59.4.814 11259626

[B8] Belelli D. Casula A. Ling A. Lambert J. J. (2002). The influence of subunit composition on the interaction of neurosteroids with GABA_A_ receptors. Neuropharmacology 43, 651–661 10.1016/S0028-3908(02)00172-7 12367610

[B9] Belelli D. Lambert J. J. (2005). Neurosteroids: endogenous regulators of the GABA_A_ receptor. Nat. Rev. Neurosci. 6, 565–575 10.1038/nrn1703 15959466

[B10] Bianchi L. Ballini C. Colivicchi M. A. Della C. L. Giovannini M. G. Pepeu G. (2003). Investigation on acetylcholine, aspartate, glutamate and GABA extracellular levels from ventral hippocampus during repeated exploratory activity in the rat. Neurochem. Res. 28, 565–573 10.1023/A:1022881625378 12675146

[B11] Brickley S. G. Cull-Candy S. G. Farrant M. (1996). Development of a tonic form of synaptic inhibition in rat cerebellar granule cells resulting from persistent activation of GABA_A_ receptors. J. Physiol. 497, 753–759 9003560 10.1113/jphysiol.1996.sp021806 PMC1160971

[B12] Brickley S. G. Mody I. (2012). Extrasynaptic GABA_A_ receptors: their function in the CNS and implications for disease. Neuron 73, 23–34 10.1016/j.neuron.2011.12.012 22243744 PMC3399243

[B13] Bright D. P. Aller M. I. Brickley S. G. (2007). Synaptic release generates a tonic GABA_A_ receptor-mediated conductance that modulates burst precision in thalamic relay neurons. J. Neurosci. 27, 2560–2569 10.1523/JNEUROSCI.5100-06.2007 17344393 PMC6672513

[B14] Bright D. P. Renzi M. Bartram J. McGee T. P. MacKenzie G. Hosie A. M. (2011). Profound desensitization by ambient GABA limits activation of δ-containing GABA_A_ receptors during spillover. J. Neurosci. 31, 753–763 10.1523/JNEUROSCI.2996-10.2011 21228184 PMC3059572

[B15] Bright D. P. Smart T. G. (2013). Protein kinase C regulates tonic GABAA receptor-mediated inhibition in the hippocampus and thalamus. Eur. J. Neurosci. 38, 3408–3423 10.1111/ejn.12352 24102973 PMC4165308

[B16] Brown N. Kerby J. Bonnert T. P. Whiting P. J. Wafford K. A. (2002). Pharmacological characterization of a novel cell line expressing human α4β3δ GABA_A_ receptors. Br. J. Pharmacol. 136, 965–974 10.1038/sj.bjp.0704795 12145096 PMC1573424

[B17] Calero C. I. Vickers E. Moraga C. G. Aguayo L. G. von G. H. Calvo D. J. (2011). Allosteric modulation of retinal GABA receptors by ascorbic acid. J. Neurosci. 31, 9672–9682 10.1523/JNEUROSCI.5157-10.2011 21715633 PMC3160198

[B18] Caraiscos V. B. Elliott E. M. You-Ten K. E. Cheng V. Y. Belelli D. Newell J. G. (2004). Tonic inhibition in mouse hippocampal CA1 pyramidal neurons is mediated by α5 subunit-containing gamma-aminobutyric acid type A receptors. Proc. Natl. Acad. Sci. U.S.A. 101, 3662–3667 10.1073/pnas.0307231101 14993607 PMC373519

[B19] Casula M. A. Bromidge F. A. Pillai G. V. Wingrove P. B. Martin K. Maubach K. (2001). Identification of amino acid residues responsible for the alpha5 subunit binding selectivity of L-655,708, a benzodiazepine binding site ligand at the GABA_A_ receptor. J. Neurochem. 77, 445–451 10.1046/j.1471-4159.2001.00289.x 11299307

[B20] Chadderton P. Margrie T. W. Hausser M. (2004). Integration of quanta in cerebellar granule cells during sensory processing. Nature 428, 856–860 10.1038/nature02442 15103377

[B21] Cope D. W. Hughes S. W. Crunelli V. (2005). GABA_A_ receptor-mediated tonic inhibition in thalamic neurons. J. Neurosci. 25, 11553–11563 10.1523/JNEUROSCI.3362-05.2005 16354913 PMC6726040

[B22] de Groote L. Linthorst A. C. (2007). Exposure to novelty and forced swimming evoke stressor-dependent changes in extracellular GABA in the rat hippocampus. Neuroscience 148, 794–805 10.1016/j.neuroscience.2007.06.030 17693036

[B23] De Koninck Y. Mody I. (1994). Noise analysis of miniature IPSCs in adult rat brain slices: properties and modulation of synaptic GABA_A_ receptor channels. J. Neurophysiol. 71, 1318–1335 8035217 10.1152/jn.1994.71.4.1318

[B24] Del Arco A. Segovia G. Fuxe K. Mora F. (2003). Changes in dialysate concentrations of glutamate and GABA in the brain: an index of volume transmission mediated actions? J. Neurochem. 85, 23–33 10.1046/j.1471-4159.2003.01692.x 12641724

[B25] Ding R. Asada H. Obata K. (1998). Changes in extracellular glutamate and GABA levels in the hippocampal CA3 and CA1 areas and the induction of glutamic acid decarboxylase-67 in dentate granule cells of rats treated with kainic acid. Brain Res. 800, 105–113 10.1016/S0006-8993(98)00507-1 9685600

[B26] Drasbek K. R. Jensen K. (2006). THIP, a hypnotic and antinociceptive drug, enhances an extrasynaptic GABA_A_ receptor-mediated conductance in mouse neocortex. Cereb. Cortex 16, 1134–1141 10.1093/cercor/bhj055 16221925

[B27] Duguid I. Branco T. London M. Chadderton P. Hausser M. (2012). Tonic inhibition enhances fidelity of sensory information transmission in the cerebellar cortex. J. Neurosci. 32, 11132–11143 10.1523/JNEUROSCI.0460-12.2012 22875944 PMC6363100

[B28] Ebert B. Wafford K. A. Whiting P. J. Krogsgaard-Larsen P. Kemp J. A. (1994). Molecular pharmacology of gamma-aminobutyric acid type A receptor agonists and partial agonists in oocytes injected with different α, β, and γ receptor subunit combinations. Mol. Pharmacol. 46, 957–963 7969086

[B29] Eghbali M. Curmi J. P. Birnir B. Gage P. W. (1997). Hippocampal GABA_A_ channel conductance increased by diazepam. Nature 388, 71–75 9214504 10.1038/40404

[B30] Erkkila B. E. Sedelnikova A. V. Weiss D. S. (2008). Stoichiometric pore mutations of the GABA_A_R reveal a pattern of hydrogen bonding with picrotoxin. Biophys. J. 94, 4299–4306 10.1529/biophysj.107.118455 18310243 PMC2480680

[B31] Farrant M. Kaila K. (2007). The cellular, molecular and ionic basis of GABA_A_ receptor signalling. Prog. Brain Res. 160, 59–87 10.1016/S0079-6123(06)60005-8 17499109

[B32] Farrant M. Nusser Z. (2005). Variations on an inhibitory theme: phasic and tonic activation of GABA_A_ receptors. Nat. Rev. Neurosci. 6, 215–229 10.1038/nrn1625 15738957

[B33] Glykys J. Mann E. O. Mody I. (2008). Which GABA_A_ receptor subunits are necessary for tonic inhibition in the hippocampus? J. Neurosci. 28, 1421–1426 10.1523/JNEUROSCI.4751-07.2008 18256262 PMC6671570

[B34] Glykys J. Mody I. (2006). Hippocampal network hyperactivity after selective reduction of tonic inhibition in GABA_A_ receptor α5 subunit-deficient mice. J. Neurophysiol. 95, 2796–2807 10.1152/jn.01122.2005 16452257

[B35] Glykys J. Mody I. (2007a). Activation of GABA_A_ receptors: views from outside the synaptic cleft. Neuron 56, 763–770 10.1016/j.neuron.2007.11.002 18054854

[B36] Glykys J. Mody I. (2007b). The main source of ambient GABA responsible for tonic inhibition in the mouse hippocampus. J. Physiol 582, 1163–1178 10.1113/jphysiol.2007.134460 17525114 PMC2075237

[B37] Hajos N. Ellender T. J. Zemankovics R. Mann E. O. Exley R. Cragg S. J. (2009). Maintaining network activity in submerged hippocampal slices: importance of oxygen supply. Eur. J. Neurosci. 29, 319–327 10.1111/j.1460-9568.2008.06577.x 19200237 PMC2695157

[B38] Hajos N. Mody I. (2009). Establishing a physiological environment for visualized *in vitro* brain slice recordings by increasing oxygen supply and modifying aCSF content. J. Neurosci. Methods 183, 107–113 10.1016/j.jneumeth.2009.06.005 19524611 PMC2753642

[B39] Hajos N. Palhalmi J. Mann E. O. Nemeth B. Paulsen O. Freund T. F. (2004). Spike timing of distinct types of GABAergic interneuron during hippocampal gamma oscillations *in vitro*. J. Neurosci. 24, 9127–9137 10.1523/JNEUROSCI.2113-04.2004 15483131 PMC6730063

[B40] Hamann M. Rossi D. J. Attwell D. (2002). Tonic and spillover inhibition of granule cells control information flow through cerebellar cortex. Neuron 33, 625–633 10.1016/S0896-6273(02)00593-7 11856535

[B41] Hartveit E. Veruki M. L. (2006). Studying properties of neurotransmitter receptors by non-stationary noise analysis of spontaneous synaptic currents. J. Physiol. 574, 751–785 10.1113/jphysiol.2006.111856 16728447 PMC1817749

[B42] Hartveit E. Veruki M. L. (2007). Studying properties of neurotransmitter receptors by non-stationary noise analysis of spontaneous postsynaptic currents and agonist-evoked responses in outside-out patches. Nat. Protoc. 2, 434–448 10.1038/nprot.2007.47 17406605

[B43] Herd M. B. Belelli D. Lambert J. J. (2007). Neurosteroid modulation of synaptic and extrasynaptic GABA_A_ receptors. Pharmacol. Ther. 116, 20–34 10.1016/j.pharmthera.2007.03.007 17531325

[B44] Herd M. B. Haythornthwaite A. R. Rosahl T. W. Wafford K. A. Homanics G. E. Lambert J. J. (2008). The expression of GABA_A_ β subunit isoforms in synaptic and extrasynaptic receptor populations of mouse dentate gyrus granule cells. J. Physiol. 586, 989–1004 10.1113/jphysiol.2007.146746 18079158 PMC2375644

[B45] Hille B. (1992). Ionic Channels of Excitable Membranes, 2nd Edn. Sunderland, MA: Sinauer Associates

[B46] Holter N. I. Zylla M. M. Zuber N. Bruehl C. Draguhn A. (2010). Tonic GABAergic control of mouse dentate granule cells during postnatal development. Eur. J. Neurosci. 32, 1300–1309 10.1111/j.1460-9568.2010.07331.x 20846322

[B47] Hosie A. M. Clarke L. da S. H. Smart T. G. (2009). Conserved site for neurosteroid modulation of GABA_A_ receptors. Neuropharmacology 56, 149–154 10.1016/j.neuropharm.2008.07.050 18762201

[B48] Hosie A. M. Dunne E. L. Harvey R. J. Smart T. G. (2003). Zinc-mediated inhibition of GABA_A_ receptors: discrete binding sites underlie subtype specificity. Nat. Neurosci. 6, 362–369 10.1038/nn1030 12640458

[B49] Hosie A. M. Wilkins M. E. da Silva H. M. Smart T. G. (2006). Endogenous neurosteroids regulate GABA_A_ receptors through two discrete transmembrane sites. Nature 444, 486–489 10.1038/nature05324 17108970

[B50] Houston C. M. Hosie A. M. Smart T. G. (2008). Distinct regulation of beta2 and beta3 subunit-containing cerebellar synaptic GABA_A_ receptors by calcium/calmodulin-dependent protein kinase II. J. Neurosci. 28, 7574–7584 10.1523/JNEUROSCI.5531-07.2008 18650335 PMC6670840

[B51] Houston C. M. McGee T. P. MacKenzie G. Troyano-Cuturi K. Rodriguez P. M. Kutsarova E. (2012). Are extrasynaptic GABA_A_ receptors important targets for sedative/hypnotic drugs? J. Neurosci. 32, 3887–3897 10.1523/JNEUROSCI.5406-11.2012 22423109 PMC4620914

[B52] Im M. S. Hamilton B. J. Carter D. B. Im W. B. (1992). Selective potentiation of GABA-mediated Cl^−^ current by lanthanum ion in subtypes of cloned GABA_A_ receptors. Neurosci. Lett. 144, 165–168 10.1016/0304-3940(92)90741-O 1279483

[B53] Inoue M. Akaike N. (1988). Blockade of gamma-aminobutyric acid-gated chloride current in frog sensory neurons by picrotoxin. Neurosci. Res. 5, 380–394 10.1016/0168-0102(88)90024-7 2456501

[B54] Irving H. Williams R. J. P. (1953). The stability of transition-metal complexes. J. Chem. Soc. 3192–3210 10.1039/jr95300031922456501

[B55] Jenkins A. Franks N. P. Lieb W. R. (1999). Effects of temperature and volatile anesthetics on GABA_A_ receptors. Anesthesiology 90, 484–491 10.1097/00000542-199902000-00024 9952156

[B56] Jensen M. L. Wafford K. A. Brown A. R. Belelli D. Lambert J. J. Mirza N. R. (2013). A study of subunit selectivity, mechanism and site of action of the δ selective compound 2 (DS2) at human recombinant and rodent native GABA_A_ receptors. Br. J. Pharmacol. 168, 1118–1132 10.1111/bph.12001 23061935 PMC3594672

[B57] Jia F. Pignataro L. Schofield C. M. Yue M. Harrison N. L. Goldstein P. A. (2005). An extrasynaptic GABA_A_ receptor mediates tonic inhibition in thalamic VB neurons. J. Neurophysiol. 94, 4491–4501 10.1152/jn.00421.2005 16162835

[B58] Kaneda M. Farrant M. Cull-Candy S. G. (1995). Whole-cell and single-channel currents activated by GABA and glycine in granule cells of the rat cerebellum. J. Physiol 485, 419–435 7545231 10.1113/jphysiol.1995.sp020739 PMC1158002

[B59] Kekesi K. A. Dobolyi A. Salfay O. Nyitrai G. Juhasz G. (1997). Slow wave sleep is accompanied by release of certain amino acids in the thalamus of cats. Neuroreport 8, 1183–1186 10.1097/00001756-199703240-00025 9175110

[B60] Kim J. A. Connors B. W. (2012). High temperatures alter physiological properties of pyramidal cells and inhibitory interneurons in hippocampus. Front. Cell Neurosci. 6:27 10.3389/fncel.2012.00027 22783167 PMC3390787

[B61] Korpi E. R. Kuner T. Seeburg P. H. Luddens H. (1995). Selective antagonist for the cerebellar granule cell-specific gamma-aminobutyric acid type A receptor. Mol. Pharmacol. 47, 283–289 7870036

[B62] Korshoej A. R. Holm M. M. Jensen K. Lambert J. D. (2010). Kinetic analysis of evoked IPSCs discloses mechanism of antagonism of synaptic GABA_A_ receptors by picrotoxin. Br. J. Pharmacol. 159, 636–649 10.1111/j.1476-5381.2009.00542.x 20105180 PMC2828027

[B62a] Krishek B. J. Moss S. J. Smart T. G. (1996). A functional comparison of the antagonists bicuculline and picrotoxin at recombinant GABAA receptors. Neuropharmacology. 35, 1289–1298 10.1016/S0028-3908(96)00089-5 9014144 10.1016/s0028-3908(96)00089-5

[B63] Krishek B. J. Moss S. J. Smart T. G. (1998). Interaction of H^+^ and Zn^2+^ on recombinant and native rat neuronal GABA_A_ receptors. J. Physiol 507(Pt 3), 639-652 10.1111/j.1469-7793.1998.639bs.x 9508826 PMC2230811

[B64] Labarrera C. London M. Angelo K. (2013). Tonic inhibition sets the state of excitability in olfactory bulb granule cells. J. Physiol. 591, 1841–1850 10.1113/jphysiol.2012.241851 23318869 PMC3624854

[B65] Lee C. Y. Liou H. H. (2013). GABAergic tonic inhibition is regulated by developmental age and epilepsy in the dentate gyrus. Neuroreport 24, 515–519 10.1097/WNR.0b013e32836205bc 23681488

[B66] Lerma J. Herranz A. S. Herreras O. Abraira V. Martin del R. R. (1986). *In vivo* determination of extracellular concentration of amino acids in the rat hippocampus. A method based on brain dialysis and computerized analysis. Brain Res. 384, 145–155 10.1016/0006-8993(86)91230-8 3790989

[B67] Lingle C. J. (2006). Empirical considerations regarding the use of ensemble-variance analysis of macroscopic currents. J. Neurosci. Methods 158, 121–132 10.1016/j.jneumeth.2006.05.027 16814867

[B68] Luscher B. Fuchs T. Kilpatrick C. L. (2011). GABA_A_ receptor trafficking-mediated plasticity of inhibitory synapses. Neuron 70, 385–409 10.1016/j.neuron.2011.03.024 21555068 PMC3093971

[B69] Machu T. K. Dillon G. H. Huang R. Lovinger D. M. Leidenheimer N. J. (2006). Temperature: an important experimental variable in studying PKC modulation of ligand-gated ion channels. Brain Res. 1086, 1–8 10.1016/j.brainres.2006.01.091 16626662

[B70] Maguire J. L. Stell B. M. Rafizadeh M. Mody I. (2005). Ovarian cycle-linked changes in GABA_A_ receptors mediating tonic inhibition alter seizure susceptibility and anxiety. Nat. Neurosci. 8, 797–804 10.1038/nn1469 15895085

[B71] Mangan P. S. Sun C. Carpenter M. Goodkin H. P. Sieghart W. Kapur J. (2005). Cultured hippocampal pyramidal neurons express two kinds of GABA_A_ receptors. Mol. Pharmacol. 67, 775–788 10.1124/mol.104.007385 15613639

[B72] McCartney M. R. Deeb T. Z. Henderson T. N. Hales T. G. (2007). Tonically active GABA_A_ receptors in hippocampal pyramidal neurons exhibit constitutive GABA-independent gating. Mol. Pharmacol. 71, 539–548 10.1124/mol.106.028597 17090706

[B73] McGee T. P. Houston C. M. Brickley S. G. (2013). Copper block of extrasynaptic GABA_A_ receptors in the mature cerebellum and striatum. J. Neurosci. 33, 13431–13435 10.1523/JNEUROSCI.1908-13.2013 23946400 PMC3742929

[B74] Millingen M. Bridle H. Jesorka A. Lincoln P. Orwar O. (2008). Ligand-specific temperature-dependent shifts in EC50 values for the GABA_A_ receptor. Anal. Chem. 80, 340–343 10.1021/ac702148p 18052252

[B75] Mitchell S. J. Silver R. A. (2000). GABA spillover from single inhibitory axons suppresses low-frequency excitatory transmission at the cerebellar glomerulus. J. Neurosci. 20, 8651–8658 11102470 10.1523/JNEUROSCI.20-23-08651.2000 PMC6773066

[B76] Mortensen M. Ebert B. Wafford K. Smart T. G. (2010). Distinct activities of GABA agonists at synaptic- and extrasynaptic-type GABA_A_ receptors. J. Physiol 588, 1251–1268 10.1113/jphysiol.2009.182444 20176630 PMC2872731

[B77] Mortensen M. Kristiansen U. Ebert B. Frolund B. Krogsgaard-Larsen P. Smart T. G. (2004). Activation of single heteromeric GABA_A_ receptor ion channels by full and partial agonists. J. Physiol. 557, 389–413 10.1113/jphysiol.2003.054734 14990676 PMC1665090

[B78] Mortensen M. Smart T. G. (2006). Extrasynaptic αβ subunit GABA_A_ receptors on rat hippocampal pyramidal neurons. J. Physiol 577, 841–856 10.1113/jphysiol.2006.117952 17023503 PMC1890388

[B79] Moss S. J. Smart T. G. (2001). Constructing inhibitory synapses. Nat. Rev. Neurosci. 2, 240–250 10.1038/35067500 11283747

[B80] Mtchedlishvili Z. Kapur J. (2006). High-affinity, slowly desensitizing GABA_A_ receptors mediate tonic inhibition in hippocampal dentate granule cells. Mol. Pharmacol. 69, 564–575 10.1124/mol.105.016683 16282519

[B81] Nani F. Bright D. P. Revilla-Sanchez R. Tretter V. Moss S. J. Smart T. G. (2013). Tyrosine phosphorylation of GABA_A_ receptor γ2-subunit regulates tonic and phasic inhibition in the thalamus. J. Neurosci. 33, 12718–12727 10.1523/JNEUROSCI.0388-13.2013 23904608 PMC4400286

[B82] Naylor D. E. Liu H. Wasterlain C. G. (2005). Trafficking of GABA_A_ receptors, loss of inhibition, and a mechanism for pharmacoresistance in status epilepticus. J. Neurosci. 25, 7724–7733 10.1523/JNEUROSCI.4944-04.2005 16120773 PMC6725248

[B83] Newland C. F. Cull-Candy S. G. (1992). On the mechanism of action of picrotoxin on GABA receptor channels in dissociated sympathetic neurones of the rat. J. Physiol 447, 191–213 1317428 10.1113/jphysiol.1992.sp018998 PMC1176032

[B84] Nusser Z. Cull-Candy S. Farrant M. (1997). Differences in synaptic GABA_A_ receptor number underlie variation in GABA mini amplitude. Neuron 19, 697–709 10.1016/S0896-6273(00)80382-7 9331359

[B85] Nusser Z. Mody I. (2002). Selective modulation of tonic and phasic inhibitions in dentate gyrus granule cells. J. Neurophysiol. 87, 2624–2628 10.1152/jn.00866.2001 11976398

[B86] Otis T. S. Mody I. (1992). Modulation of decay kinetics and frequency of GABA_A_ receptor-mediated spontaneous inhibitory postsynaptic currents in hippocampal neurons. Neuroscience 49, 13–32 10.1016/0306-4522(92)90073-B 1357584

[B87] Pan Z. H. Bahring R. Grantyn R. Lipton S. A. (1995). Differential modulation by sulfhydryl redox agents and glutathione of GABA- and glycine-evoked currents in rat retinal ganglion cells. J. Neurosci. 15, 1384–1391 7869105 10.1523/JNEUROSCI.15-02-01384.1995 PMC6577822

[B88] Park J. B. Skalska S. Son S. Stern J. E. (2007). Dual GABA_A_ receptor-mediated inhibition in rat presympathetic paraventricular nucleus neurons. J. Physiol 582, 539–551 10.1113/jphysiol.2007.133223 17495040 PMC2075349

[B89] Peden D. R. Petitjean C. M. Herd M. B. Durakoglugil M. S. Rosahl T. W. Wafford K. Lambert J. J. (2008). Developmental maturation of synaptic and extrasynaptic GABA_A_ receptors in mouse thalamic ventrobasal neurones. J. Physiol. 586, 965–987 10.1113/jphysiol.2007.145375 18063661 PMC2375643

[B90] Perrais D. Ropert N. (1999). Effect of zolpidem on miniature IPSCs and occupancy of postsynaptic GABA_A_ receptors in central synapses. J. Neurosci. 19, 578–588 9880578 10.1523/JNEUROSCI.19-02-00578.1999 PMC6782193

[B91] Porcello D. M. Huntsman M. M. Mihalek R. M. Homanics G. E. Huguenard J. R. (2003). Intact synaptic GABAergic inhibition and altered neurosteroid modulation of thalamic relay neurons in mice lacking delta subunit. J. Neurophysiol. 89, 1378–1386 10.1152/jn.00899.2002 12626617

[B92] Quirk K. Blurton P. Fletcher S. Leeson P. Tang F. Mellilo D. (1996). [3H]L-655,708, a novel ligand selective for the benzodiazepine site of GABA_A_ receptors which contain the α5 subunit. Neuropharmacology 35, 1331–1335 10.1016/S0028-3908(96)00061-5 9014149

[B93] Richardson B. D. Ling L. L. Uteshev V. V. Caspary D. M. (2013). Reduced GABA_A_ receptor-mediated tonic inhibition in aged rat auditory thalamus. J. Neurosci. 33, 1218a–1227a 10.1523/JNEUROSCI.3277-12.2013 23325258 PMC3717293

[B94] Saliba R. S. Kretschmannova K. Moss S. J. (2012). Activity-dependent phosphorylation of GABA_A_ receptors regulates receptor insertion and tonic current. EMBO J. 31, 2937–2951 10.1038/emboj.2012.109 22531784 PMC3395084

[B95] Saxena N. C. Neelands T. R. Macdonald R. L. (1997). Contrasting actions of lanthanum on different recombinant gamma-aminobutyric acid receptor isoforms expressed in L929 fibroblasts. Mol. Pharmacol. 51, 328–335 9203639 10.1124/mol.51.2.328

[B96] Scimemi A. Semyanov A. Sperk G. Kullmann D. M. Walker M. C. (2005). Multiple and plastic receptors mediate tonic GABA_A_ receptor currents in the hippocampus. J. Neurosci. 25, 10016–10024 10.1523/JNEUROSCI.2520-05.2005 16251450 PMC6725560

[B97] Sedelnikova A. Erkkila B. E. Harris H. Zakharkin S. O. Weiss D. S. (2006). Stoichiometry of a pore mutation that abolishes picrotoxin-mediated antagonism of the GABA_A_ receptor. J. Physiol 577, 569–577 10.1113/jphysiol.2006.120287 16990398 PMC1890441

[B98] Seutin V. Johnson S. W. (1999). Recent advances in the pharmacology of quaternary salts of bicuculline. Trends Pharmacol. Sci. 20, 268–270 10.1016/S0165-6147(99)01334-6 10390643

[B99] Shen H. Gong Q. H. Yuan M. Smith S. S. (2005). Short-term steroid treatment increases delta GABA_A_ receptor subunit expression in rat CA1 hippocampus: pharmacological and behavioral effects. Neuropharmacology 49, 573–586 10.1016/j.neuropharm.2005.04.026 15950994 PMC2887348

[B100] Sigworth F. J. (1980). The variance of sodium current fluctuations at the node of Ranvier. J. Physiol 307, 97–129 6259340 10.1113/jphysiol.1980.sp013426 PMC1283036

[B101] Smart T. G. Constanti A. (1982). A novel effect of zinc on the lobster muscle GABA receptor. Proc. R. Soc. Lond B Biol. Sci. 215, 327–341 10.1098/rspb.1982.0045 6127710

[B102] Smart T. G. Constanti A. (1986). Studies on the mechanism of action of picrotoxinin and other convulsants at the crustacean muscle GABA receptor. Proc. Roy. Soc. Lond. B Biol. Sci. 227, 191–216 10.1098/rspb.1986.0019 26151987

[B103] Song I. Savtchenko L. Semyanov A. (2011). Tonic excitation or inhibition is set by GABA_A_ conductance in hippocampal interneurons. Nat. Commun. 2, 376 10.1038/ncomms1377 21730957 PMC3144593

[B104] Stell B. M. Brickley S. G. Tang C. Y. Farrant M. Mody I. (2003). Neuroactive steroids reduce neuronal excitability by selectively enhancing tonic inhibition mediated by δ subunit-containing GABA_A_ receptors. Proc. Natl. Acad. Sci. U.S.A. 100, 14439–14444 10.1073/pnas.2435457100 14623958 PMC283610

[B105] Stell B. M. Mody I. (2002). Receptors with different affinities mediate phasic and tonic GABA_A_ conductances in hippocampal neurons. J. Neurosci. 22, RC223 12006605 10.1523/JNEUROSCI.22-10-j0003.2002 PMC6757628

[B106] Storustovu S. I. Ebert B. (2006). Pharmacological characterization of agonists at delta-containing GABA_A_ receptors: Functional selectivity for extrasynaptic receptors is dependent on the absence of gamma2. J. Pharmacol. Exp. Ther. 316, 1351–1359 10.1124/jpet.105.092403 16272218

[B107] Takeuchi A. Takeuchi N. (1969). A study of the action of picrotoxin on the inhibitory neuromuscular junction of the crayfish. J. Physiol 205, 377–391 5357245 10.1113/jphysiol.1969.sp008972 PMC1348609

[B108] Tang X. Hernandez C. C. Macdonald R. L. (2010). Modulation of spontaneous and GABA-evoked tonic α4β3δ and α4β3γ2L GABA_A_ receptor currents by protein kinase A. J. Neurophysiol. 103, 1007–1019 10.1152/jn.00801.2009 19939957 PMC2822691

[B109] Thompson S. M. Masukawa L. M. Prince D. A. (1985). Temperature dependence of intrinsic membrane properties and synaptic potentials in hippocampal CA1 neurons *in vitro*. J. Neurosci. 5, 817–824 3973697 10.1523/JNEUROSCI.05-03-00817.1985 PMC6565032

[B110] Tia S. Wang J. F. Kotchabhakdi N. Vicini S. (1996). Developmental changes of inhibitory synaptic currents in cerebellar granule neurons: role of GABA_A_ receptor α6 subunit. J. Neurosci. 16, 3630–3640 8642407 10.1523/JNEUROSCI.16-11-03630.1996 PMC6578841

[B111] Timmerman W. Westerink B. H. (1997). Brain microdialysis of GABA and glutamate: what does it signify? Synapse 27, 242–261 10.1002/(SICI)1098-2396(199711)27:3<242::AID-SYN9>3.0.CO;2-D 9329159

[B112] Tossman U. Jonsson G. Ungerstedt U. (1986). Regional distribution and extracellular levels of amino acids in rat central nervous system. Acta Physiol. Scand. 127, 533–545 10.1111/j.1748-1716.1986.tb07938.x 2875604

[B113] Traynelis S. F. Jaramillo F. (1998). Getting the most out of noise in the central nervous system. Trends Neurosci. 21, 137–145 10.1016/S0166-2236(98)01238-7 9554720

[B114] Trevelyan A. J. Jack J. (2002). Detailed passive cable models of layer 2/3 pyramidal cells in rat visual cortex at different temperatures. J. Physiol 539, 623–636 10.1113/jphysiol.2001.013291 11882693 PMC2290153

[B115] Ueno S. Bracamontes J. Zorumski C. Weiss D. S. Steinbach J. H. (1997). Bicuculline and gabazine are allosteric inhibitors of channel opening of the GABA_A_ receptor. J. Neurosci. 17, 625–634 8987785 10.1523/JNEUROSCI.17-02-00625.1997 PMC6573228

[B116] van der Zeyden M. Oldenziel W. H. Rea K. Cremers T. I. Westerink B. H. (2008). Microdialysis of GABA and glutamate: analysis, interpretation and comparison with microsensors. Pharmacol. Biochem. Behav. 90, 135–147 10.1016/j.pbb.2007.09.004 17939932

[B117] Vanini G. Wathen B. L. Lydic R. Baghdoyan H. A. (2011). Endogenous GABA levels in the pontine reticular formation are greater during wakefulness than during rapid eye movement sleep. J. Neurosci. 31, 2649–2656 10.1523/JNEUROSCI.5674-10.2011 21325533 PMC3073841

[B118] Volgushev M. Vidyasagar T. R. Chistiakova M. Yousef T. Eysel U. T. (2000). Membrane properties and spike generation in rat visual cortical cells during reversible cooling. J. Physiol 522(Pt 1), 59–76 10.1111/j.1469-7793.2000.0059m.x 10618152 PMC2269736

[B119] Wafford K. A. Thompson S. A. Thomas D. Sikela J. Wilcox A. S. Whiting P. J. (1996). Functional characterization of human γ-aminobutyric acid_*A*_ receptors containing the α4 subunit. Mol. Pharmacol. 50, 670–678 8794909

[B120] Wafford K. A. van Niel M. B. Ma Q. P. Horridge E. Herd M. B. Peden D. R. Lambert J. J. (2009). Novel compounds selectively enhance delta subunit containing GABA_A_ receptors and increase tonic currents in thalamus. Neuropharmacology 56, 182–189 10.1016/j.neuropharm.2008.08.004 18762200

[B121] Wall M. J. (2002). Furosemide reveals heterogeneous GABA_A_ receptor expression at adult rat Golgi cell to granule cell synapses. Neuropharmacology 43, 737–749 10.1016/S0028-3908(02)00085-0 12367619

[B122] Wei W. Zhang N. Peng Z. Houser C. R. Mody I. (2003). Perisynaptic localization of delta subunit-containing GABA_A_ receptors and their activation by GABA spillover in the mouse dentate gyrus. J. Neurosci. 23, 10650–10661 14627650 10.1523/JNEUROSCI.23-33-10650.2003 PMC6740905

[B123] Wlodarczyk A. I. Sylantyev S. Herd M. B. Kersante F. Lambert J. J. Rusakov D. A. (2013). GABA-independent GABA_A_ receptor openings maintain tonic currents. J. Neurosci. 33, 3905–3914 10.1523/JNEUROSCI.4193-12.2013 23447601 PMC3591781

[B124] Wohlfarth K. M. Bianchi M. T. MacDonald R. L. (2002). Enhanced neurosteroid potentiation of ternary GABA_A_ receptors containing the delta subunit. J. Neurosci. 22, 1541–1549 11880484 10.1523/JNEUROSCI.22-05-01541.2002 PMC6758857

[B125] Xi Z. X. Ramamoorthy S. Shen H. Lake R. Samuvel D. J. Kalivas P. W. (2003). GABA transmission in the nucleus accumbens is altered after withdrawal from repeated cocaine. J. Neurosci. 23, 3498–3505 12716959 10.1523/JNEUROSCI.23-08-03498.2003 PMC6742318

[B126] Yamada J. Furukawa T. Ueno S. Yamamoto S. Fukuda A. (2007). Molecular basis for the GABA_A_ receptor-mediated tonic inhibition in rat somatosensory cortex. Cereb. Cortex 17, 1782–1787 10.1093/cercor/bhl087 16997904

[B127] Zhu W. J. Wang J. F. Corsi L. Vicini S. (1998). Lanthanum-mediated modification of GABA_A_ receptor deactivation, desensitization and inhibitory synaptic currents in rat cerebellar neurons. J. Physiol. 511, 647–661 10.1111/j.1469-7793.1998.647bg.x 9714849 PMC2231154

